# Value-based genomic screening: exploring genomic screening for chronic diseases using triple value principles

**DOI:** 10.1186/s12913-019-4703-z

**Published:** 2019-11-11

**Authors:** Viktor Dombrádi, Erica Pitini, Carla G. van El, Anant Jani, Martina Cornel, Paolo Villari, Muir Gray, Klára Bíró

**Affiliations:** 1 Department of Health Systems Management and Quality Management for Health Care, Faculty of Public Health, University of Debrecen, Debrecen, Hungary; 2 Department of Public Health and Infectious Diseases, Sapienza University of Rome, Rome, Italy; 3 Department of Clinical Genetics/Amsterdam Public Health research Institute, Section Community Genetics, Amsterdam UMC, Vrije Universiteit, Amsterdam, The Netherlands; 4 Value Based Healthcare Programme, Department of Primary Care, University of Oxford, Oxford, UK; 5 Oxford Centre for Triple Value Healthcare, Oxford, UK

**Keywords:** Value-based healthcare, Genomics, Screening, Prevention, Chronic disease

## Abstract

**Background:**

Genomic screening has unique challenges which makes it difficult to easily implement on a wide scale. If the costs, benefits and tradeoffs of investing in genomic screening are not evaluated properly, there is a risk of wasting finite healthcare resources and also causing avoidable harm.

**Main text:**

If healthcare professionals – including policy makers, payers and providers – wish to incorporate genomic screening into healthcare while minimizing waste, maximizing benefits, and considering results that matter to patients, using the principles of triple value (allocative, technical, and personal value) could help them to evaluate tough decisions and tradeoffs. Allocative value focuses on the optimal distribution of limited healthcare resources to maximize the health benefits to the entire population while also accounting for all the costs of care delivery. Technical value ensures that for any given condition, the right intervention is chosen and delivered in the right way. Various methods (e.g. ACCE, HTA, and Wilson and Jungner screening criteria) exist that can help identify appropriate genomic applications. Personal value incorporates preference based informed decision making to ensure that patients are informed about the benefits and harms of the choices available to them and to ensure they make choices based on their values and preferences.

**Conclusions:**

Using triple value principles can help healthcare professionals make reasoned and tough judgements about benefits and tradeoffs when they are exploring the role genomic screening for chronic diseases could play in improving the health of their patients and populations.

## Background

### History and hype of genomics

The Human Genome Project began in 1990 with the goal to map the entire human genome [1]. In April 2003 the project was declared complete and ~ 22,300 genes were identified with 99.99% accuracy. Although the function of these genes was not explored in the project, hopes were high regarding how this new technology would revolutionise medicine. In retrospect, many experts claim that there was too much hype surrounding the topic [2, 3]. In April 2005, 2 years after the Human Genome Project was completed, a group of international researchers met for 1 week in Bellagio, Italy, to discuss how healthcare could benefit from the human genome [4]. During the meeting various obstacles were identified that hinder the usage of genomic technology in everyday clinical practice. For example, most genetic diseases are polygenic (oligogenic), and thus it is very challenging to predict if and when these manifest during an individual’s lifetime [2]. Also, in some instances – such as Huntington’s disease – the genes associated with the disease can be identified, but besides preimplantation genetic diagnosis to prevent disease manifesting in offspring, at that time it was considered that no meaningful treatment or preventive action could be taken [5]. Over a decade has passed since the meeting and although the cost of genomic sequencing has dropped substantially [6], the same questions of how to bring genomic technology closer to healthcare and thus, promote personalized medicine, still remain [7–9].

Although genomic technology is used for various purposes in healthcare, such as pharmacogenomics, this paper will focus on genomic screening of some specific subforms of chronic diseases due to the prevalence and cost associated with these types of diseases. For example, in the United States about 50% of adults have at least one chronic disease [10] which accounts for more than 80% of annual health care expenditure [11]. Further to this, within the realm of genomic applications, the evidence base around using genomic screening for some monogenic subforms of chronic conditions is relatively more established than its use for other conditions [12]; for example, the use of cascade screening after finding index cases has proven clinical utility.

It should be noted in this paper we use the term ‘genomics’, instead of ‘genetics’, to refer to screening for single and multiple genes because genomics is a broader term and techniques may range from single gene testing and genomic sequencing for analysis of single genes to techniques utilizing gene panels, such as oncopanels. The analysis presented in this paper is based on the targeted sequencing and/or analysis of the above-mentioned monogenic subsets.

### Current usage of genomic screening for chronic diseases

Currently, genomic screening is utilized for monogenic subforms of some common chronic disorders with a complex etiology typically including gene-gene and gene-environment interaction. For instance, in cancer and cardiovascular disorders, genomic screening can be used for the detection of hereditary breast and ovarian cancer (HBOC), hereditary forms of colorectal cancer (CRC), and familial hypercholesterolemia (FH). In such cases, genomic information is mainly oriented to disease diagnosis, prognosis and individualised treatment/management and allows for prevention in family members [12]. Rather than opting for population screening for these conditions, cascade screening of first degree family members of index cases has proven to be (cost) effective for the aforementioned conditions and is, therefore, recommended by prominent international bodies. Various strategies can be applied to identify index patients including (combinations of) investigating clinical features and family history. While several definitions of ‘screening’ exist, in the context of this paper we focus on (i) systematic programmatic approaches delivered for (ii) the benefit of healthy persons. Regarding CRC, the Evaluation of Genomic Applications in Practice and Prevention (EGAPP) working group (facilitated by the United States Centers for Disease Control and Prevention Office of Public Health Genomics) recommends to routinely investigate tumor tissue of all newly diagnosed CRC cases for markers suggestive of Lynch syndrome. Genetic testing can be offered to confirm diagnosis and reduce morbidity and mortality in relatives through cascade screening and surveillance [13]. A recent systematic review identified examples of structured and permanent programs of this kind in the USA and Switzerland [14].

In the case of HBOC, the US Preventive Services Task Force recommends the use of family history screening tools by primary care providers to identify women at increased risk of developing breast cancer because of harmful mutations. Women with positive screening results are offered genetic counseling and, if indicated, offered breast cancer susceptibility genes (BRCA) testing, in order to reduce morbidity and mortality from breast and ovarian cancer [15]. Programs of this kind were implemented in various countries, such as Georgia, Italy (Emilia-Romagna Region) and Canada (Ontario) [16, 17].

In FH, finding index cases may be based on clinical symptoms combined with systematically surveying family history after which a DNA test can confirm diagnosis. The UK National Institute for Health and Care Excellence (NICE) recommends cascade (DNA) testing to identify at-risk relatives of individuals with a genetic diagnosis of FH [18]. The most famous example of such a cascade screening program existed in the Netherlands, where a national government subsidized program was active between 1994 and 2014 [19].

However, despite progress in the field of genomic screening for chronic disease, such genomic screening programs with established evidence bases combining case finding and cascade screening are not implemented on a wide scale and there is ambiguity on which tests to use because the ability to detect a mutation is in itself not enough to warrant screening [20].

## Main text

### Exploring genomic screening using triple value principles

Value-based healthcare addresses the question of how to provide results that matter to patients while optimizing the usage of limited resources [21–23]. This question gained even more importance after the 2008 financial crisis which resulted in austerities within healthcare systems internationally [24, 25]. Among many responses, the Triple Value Healthcare principles were introduced in 2010 and are currently used within the English National Health Service (NHS) RightCare Programme, the Prudent Healthcare Programme in Wales, the Realistic Medicine Programme in Scotland, the Model of Care in Saudi Arabia and have also been adopted by an EU Commission Working Group on value based healthcare [26–28]. They adapt Avedis Donabedian’s work and apply them to universal healthcare systems which have the two constraints of needing to deliver care to an entire population and doing so within a finite budget, neither of which are constraints in the US where Donabedian did most of his work [29]. Triple Value Healthcare looks at value from three perspectives (patients, populations and interventions) yielding the three components of triple value healthcare: (i) Allocative Value (mostly applicable for policy makers and payers): ensuring finite resources are allocated to deliver maximum benefit to the population being served; (ii) Technical Value (mostly applicable for providers and payers): ensuring that the interventions being used deliver the best outcomes for the resources being utilised; (iii) Personal Value (mostly applicable for clinicians within provider organisations): ensuring that patient’s objective clinical needs and subjective needs are met by the care they receive. Although these principles were originally developed for healthcare systems, the principles are also applicable to broader intervention modalities like genomic screening. In the following sections we will explore how the Triple Value Healthcare principles can help healthcare professionals make reasoned and tough judgements about the role genomic screening for chronic diseases could play for their patients and populations.

### Allocative value of genomic screening

Every decision regarding the allocation of finite resources that policy makers and payers have to make has an opportunity cost, which means that every unit of resource spent on one area within healthcare is the same unit of resource that cannot be spent elsewhere. Thus, when allocating resources, policy makers and payers must focus on maximizing the health benefits at a population level for a given unit of resource while also taking consideration of those who are most vulnerable in any given population and who may not be receiving care [30]. This means that whenever an area of waste – with waste defined as [31] “spending on services that lack evidence of producing better health outcomes compared to less-expensive alternatives; inefficiencies in the provision of health care goods and services; and costs incurred while treating avoidable medical injuries, such as preventable infections in hospitals.” – is identified, we must disinvest from it and use the liberated resources to fund more beneficial interventions, including innovation. In the context of genomic screening, one category of interventions which have questionable benefit and could be deemed as wasteful are direct to consumer personal genome testing. As highlighted by McGuire and Burke: “Similar to other screening tests-or procedures of questionable clinical value that have been marketed direct-to-consumer, such as whole body CT scans, ordering follow-up tests and providing treatment on the basis of direct-to-consumer personal genome tests of indeterminate clinical value constitutes a raid on the medical commons [32].” It is the responsibility of policy makers, payers and providers to intervene when interventions like this put a burden on already limited resources and also pose a threat by potentially undermining the validity of genomic screening as a category of intervention in the minds of patients who are sometimes misled and given false hopes about what they can achieve.

**Fig. 1:**
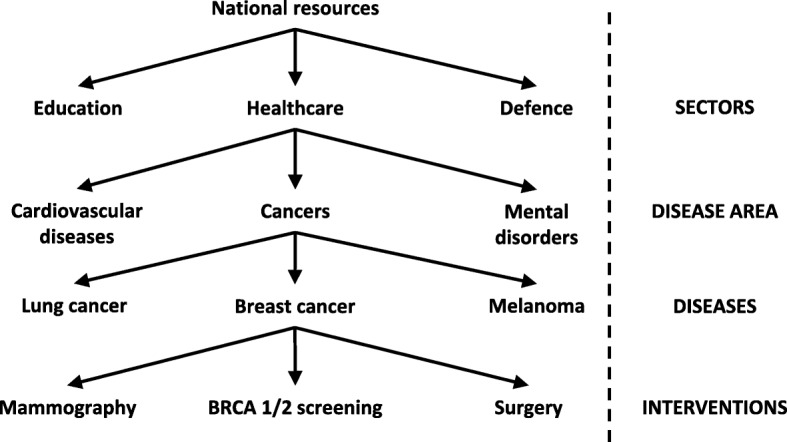
Illustration of the allocative value within the Triple Value Healthcare principles. The arrows represent the allocation of resources to lower levels

From a resource allocation perspective, Triple Value Healthcare principles promote allocation based on the disease burden within a population rather than on organizational silos such as primary care or secondary care (Fig. 1). This approach puts the population’s need at the forefront of resource allocation decisions and bypasses political and power struggles that are often times seen between different healthcare silos.

Once the budget for a disease area is determined, allocation for interventions within the disease area, including genomic testing, requires an understanding of the costs and benefits of the medical interventions currently available – i.e. technical value. This can be determined through a variety of methods (see section below on Technical Value) including using the ACCE (analytic validity, clinical validity, clinical utility, and ethical, legal, and social implications) framework, Health Technology Assessment (HTA) and use of the Wilson and Junger screening criteria for the available interventions; thus, giving a standardized method to understand the relative costs and benefits of available interventions. Furthermore, the entire care pathway costs must be considered to ensure adequate resources are available to provide the full complement of care needed.

For genomic disorders, a subset of genes is associated with multiple diseases (e.g. BRCA 1/2 is associated with breast and ovarian cancers, Lynch syndrome is generally associated with a younger age of onset for colorectal, endometrial and ovarian cancers) so when deciding how much resource should be spent on breast cancer and ovarian cancer, for example, some overlaps must be considered and the two disciplines have to come to agreement on how to fund BRCA 1/2 testing together with potentially pooled budgets. Furthermore, for genomic screenings to be meaningful, the follow up procedures contained with the entire care pathway must be funded as well – a more valid example, as compared to direct to consumer personal genomic testing, of needing to draw from the ‘medical commons’. For example, increased screening for Lynch syndrome will lead to increased confirmed cases of mutation carriers and this will also increase the number of individuals that need follow up colonoscopies; increased colonoscopies will also increase the confirmed cases of colorectal cancers, thus, resulting in the need for more surgery. This single example highlights the challenges health policy makers must consider when introducing genomic screening into any healthcare system.

### Technical value of genomic screening

It is not enough to only focus on how limited healthcare resources should be allocated because even if these resources are distributed optimally, they provide limited benefits if providers use and payers pay for the wrong interventions, if the right interventions are not executed properly by the provider or if the provider delivers the intervention to the wrong patient. The question of what factors determine the value of genomic applications has been debated for a long time and according to recent literature reviews, the ACCE framework and modified versions of HTA and the Wilson and Jungner screening criteria are the most common methods used by the scientific community to address this question [33, 34]. While the ACCE framework was developed specifically for the evaluation of genetic tests, the other approaches were developed to cover health technologies in general and then adapted or successfully applied to the evaluation of genomic technologies. Below we address the strengths and weaknesses of these approaches from a technical value lense.

The original ACCE framework has three components which are built upon each other while the fourth one permeates the first three [35]: (i) analytical validity (i.e. a test’s accuracy and reliability to measure the genotype of interest); (ii) clinical validity (i.e. how well a test can detect or predict the investigated disorder); (iii) clinical utility. There is an ongoing debate on how to define clinical utility. In its narrowest sense, clinical utility is usually defined as the improvement in health outcomes due to the test use and subsequent clinical interventions. Nevertheless, the concept of clinical utility could be extended to any use of test results to guide the clinical management of an individual with a diagnosed disorder or, further, to the improvement of any outcome considered important to the patients and their families, even if not strictly health related [36]. Moreover, the ACCE framework deepens the concept of clinical utility including considerations on contextual or implementation issues, such as cost-effectiveness, education, monitoring and evaluation. Finally, (iv) an analysis taking ethical, legal and social issues (ELSI) into account may, for instance, study effects on family dynamics, psychological well-being, influence of the test result on lifestyle, stigmatization, equity of access, and respect for privacy [37], allowing a weighing of potential harm and waste versus potential benefits of a genomic test in a specific context. The ACCE model has been adopted and adapted by various entities since its development, both in the United States and worldwide. A substantial step in the ACCE’s evolution came in 2004, when the EGAPP (Evaluation of Genomic Applications in Practice and Prevention) initiative began to leverage the ACCE model structure and experience and developed an initiative that supported the systematic assessment of genetic tests using the ACCE criteria as well as making recommendations for their use in clinical and public health practice [38].

HTA is a systematic approach for the evaluation of all health technologies, including genomic tests. Like the ACCE framework, HTA examines the properties and effects of a health technology, taking into consideration scientific, technological, medical, social, legal and ethical issues [39]. However, the peculiarity of HTA is the great attention paid to the delivery of a health technology, reflected by an in-depth analysis of the economic and organizational aspects related to its implementation [40]. An example of HTA applied to the evaluation of genomic technologies is the framework developed by McMaster University to guide public coverage of new predictive genetic tests in Ontario [41]. The U.S. Preventive Services Task Force instead, uses the same HTA methodology developed for preventive services (i.e. screening, counselling, and preventive medications) for evaluating genomic tests [42].

Finally, the Wilson and Jungner screening principles, developed under the mandate of the World Health Organization, have been applied to the evaluation of genetic screening programs; they consider different factors such as the ability of the test to detect a clinical condition, the availability of an accepted and effective treatment, and the cost-effectiveness of the screening program [33, 43].

Despite having several approaches to assess the value of specific genomic screening applications, there is no general agreement on which one should be used to address the full spectrum of evaluation questions related to new interventions. Thus, in order for providers and payers to determine whether to allocate resources to a genomic screening application and to decide if this reaches the threshold to be worth investing in, a combination of the ACCE framework, which is well suited to genetic tests, and the HTA approach, which addresses important aspects of service delivery, would be the most adequate strategy based on the findings thus far [33].

### Personal value of genomic screening

Besides the allocation of limited resources and understanding the benefits and limitations of genomic applications, clinicians within provider organisations must also consider the expectations and values of the patient. To address this, the Triple Value Healthcare principles emphasise both patient satisfaction and the active involvement of the patient through preference-based informed decision making.

As with any other healthcare services, in order to provide the best experience for patients, clinicians using genomic screening should take into consideration the following eight principles originally proposed by the Picker Institute: (i) access to care, (ii) respect for patients’ values, preferences and expressed needs, (iii) coordination and integration of care, (iv) information, communication and education, (v) physical comfort, (vi) emotional support and alleviation of fear and anxiety, (vii) involvement of family and friends, and (viii) continuity and transition [44, 45]. Beyond these general principles one must also consider the sensitive nature of the results derived from genomic testing. Thus, when communicating results regarding a high risk for a serious disorder, it is advised that either the genetic counsellor or other competent health professional gives this information in person or at least via telephone [46].

Because patients will experience the consequences, whether good or bad, of any kind of medical intervention, they have a moral right to be involved in the decision-making process. In addition, taking account of the needs, preferences and values of the patient could lead to more optimal use of resources, especially when unnecessary interventions are avoided. However, due to the limited resources available, intervention options have to be constrained within reasonable boundaries. Thus, to successfully utilize preference-based informed decision making, three factors are needed: (i) evidence of an intervention’s benefits and harms, (ii) knowledge of the individuals’ clinical condition and (iii) the values of the patient [27, 47].

The first factor is encapsulated in Technical Value. Thus, when there is no proof for the benefit of a genomic application, or if there is reliable proof that this application does not meet expectations, then it should not be offered as an option to the patient.

The second factor would normally only apply for the patient, however, in genomic screening the condition of family members, especially first degree relatives in case of dominant disorders, can be related to that of the index patient. For example, when someone is diagnosed with breast cancer, a BRCA 1/2 test has benefit for the index patient because though the disease is already manifest, it can help in selecting appropriate treatment and interventions and can predict the probability of the cancer reemerging in the future [48]. Also, for the individual whose family member has the BRCA 1/2 mutation or has a family history of breast cancer, genetic testing for BRCA 1/2 is potentially of high value, since a positive test result may lead to early surveillance.

Due to the subjective nature of personal values and preferences, generalizing is challenging but it is possible for clinicians to identify values and preferences that can be considered. For example, in the case of testing for BRCA1/2 related breast cancer, prevention or health gain would be a valued aim. Planning of pregnancies and reproductive decisions can also be based on this information; some prospective parents may want to reduce the chances of passing a disorder to the next generation and may opt for prenatal diagnosis or preimplantation genetic diagnosis (PGD), depending on their personal values [49]. People that expect not to benefit from testing for such genetic disorders can forego testing, for instance because they prefer not to know and value an open future or would not be able to cope with the test result. In all cases, personal values need to be the basis of a process of preference based informed decision making regarding genetic testing.

### Value and next generation sequencing

In recent years, the introduction of Next Generation Sequencing (NGS) has generated a further complexity in weighing the pros and cons of genomic screening. The increased use of sequencing can improve diagnosis, however, the generation of Secondary Findings (SF) and Variants of Uncertain Significance (VUS) raises ethical, legal and social issues e.g. regarding consent and use of resources. For example, for cases where clinical sequencing is used, the American College of Medical Genetics (ACMG) has recommended a deliberate search for SF in 59 genes mostly unrelated to the health problem the patient presents with [50]. This might be regarded as opportunistic screening that optimizes the use of resources that have already been utilized but critical appraisals have pointed out that this approach runs counter to many standard premises in screening [51]. A major problem related to technical value is the variable evidence related to the different genes, especially regarding the penetrance and pathogenicity of variants in an unaffected population. In Europe, the European Society of Human Genetics (ESHG) has opted for a targeted approach [52]. After sequencing analysis can be targeted to the specific genes related to monogenic subforms of common disorders, such as FH, Lynch syndrome or BRCA-related breast cancer, similarly limited panels can be used, such as an onco panel in families where there is potentially an inherited tumor syndrome.

Professional societies and health authorities are currently implicated in decisions on which genes and gene variants to report as SF on specific grounds [53]. For instance, the French Society of Predictive and Personalized Medicine (SFMPP) elaborated guidelines for managing information on SFs for cancer related genes. “The main criteria were the ‘actionability’ of the genes (available screening or prevention strategies), the risk evaluation (severity, penetrance, and age of disease onset), and the level of evidence from published data” [54]. The selected genes only partially overlapped with the ACMG oncology genes. While technical value focussing on the level of evidence is essential, prioritisation based on, for instance severity and penetrance, as well as on available resources for analysis, counselling and follow-up may inform national and local strategies to select specific subsets of genes in order to allocate resources proportionally. This typically is relevant in universal healthcare systems responsible for finite budgets. For instance in the UK’s 100,000 Genomes Project, a much more limited set of secondary findings has been selected in genes predisposing to bowel cancer, breast cancer and FH [55]. To assess allocative value, the resources devoted for people found to be a carrier of a mutation in SF without clinical symptoms would have to be evaluated. Resources spent on this group might be spent better in, for example, first degree relatives of BRCA1-carriers who are at 50% risk, while the population may be at < 1% risk. In some countries with universal health care systems, tests not subsidized by the healthcare service, can be available in the private sector so that tests with potential personal value to people are still available. Health authorities should make an effort to regulate appropriate information provision on the potential benefits and limitations of such testing to the public.

## Conclusions

Due to the limited resources available and the increasing need to provide patient-centered care, healthcare as a whole must strive to reduce waste, maximize benefits and provide results that matters to patients. Genomic screening for chronic diseases, as a broad intervention modality, must also pursue these endeavors. By taking triple value principles into consideration, healthcare professionals, payers and providers can make reasoned and tough decisions about the role genomic screening for chronic diseases can play to improve the value of healthcare delivered to their patients and populations.

## Data Availability

Not applicable.

## Abbreviations

ACCE: Analytic validity, Clinical validity, Clinical utility, and Ethical, legal, and social implications
ACMG: American College of Medical Genetics
BRCA: Breast cancer susceptibility genes
CRC: Hereditary forms of colorectal cancer
CT: Computed tomography
DNA: Deoxyribonucleic acid
EGAPP: Evaluation of Genomic Applications in Practice and Prevention
ELSI: Ethical, legal and social issues
ESHG: European Society of Human Genetics
FH: Familial hypercholesterolemia
HBOC: Hereditary breast and ovarian cancer
HTA: Health Technology Assessment
NGS: Next Generation Sequencing
NHS: National Health Service
NICE: National Institute for Health and Care Excellence
PGD: Preimplantation genetic diagnosis
SF: Secondary Findings
SFMPP: French Society of Predictive and Personalized Medicine
VUS: Variants of Uncertain Significance
